# Improving perceptions of aging and dementia using creative storytelling: implications for pedagogy

**DOI:** 10.1093/geroni/igaf122.2203

**Published:** 2025-12-31

**Authors:** Melannie Pate, Kent Guion

**Affiliations:** The University of North Carolina at Wilmington, Wilmington, North Carolina, United States; The University of North Carolina at Wilmington, Wilmington, North Carolina, United States

## Abstract

**Background:**

Negative stereotypes and ageism toward older adults with dementia remain a problem, proving to reduce quality of life and can contribute to negative health outcomes. Depictions of dementia are often associated with negative stereotypes. Training an adequate workforce for this population is essential as there is concern with the attitudes and knowledge that students and future public health professionals hold about individuals living with dementia.

**Description:**

Using TimeSlips, a creative storytelling method, undergraduate and graduate students participated in community sites serving individuals living with dementia. Students facilitated the creative storytelling sessions weekly at each site. Pre and post-evaluations measuring perceptions, outcomes, satisfaction, and knowledge were administered.

**Lessons Learned:**

• Results of the evaluation provide opportunities to view the importance of field work for students with individuals living with dementia • Creative engagement lessens negative stereotypes for students • Students enjoy undergraduate and graduate aging courses more and feel more positively about older adults when they participate in community-based field work • Pedagogical approaches that include field work to reduce negative stereotypes around aging and dementia are needed Implications • Lead to a greater understanding of students’ attitudes toward aging and dementia • Inform educational interventions to improve quality of life • Extend creative storytelling as part of the pedagogy for courses in aging and public health • Improve perceptions of dementia, resulting in less resistance and fear of working with the population

